# Hedinger Syndrome: A Rare Cardiac Manifestation of Carcinoid Syndrome

**DOI:** 10.7759/cureus.26528

**Published:** 2022-07-03

**Authors:** Hayk Ghukasyan

**Affiliations:** 1 Internal Medicine, University of Hawaii, Honolulu, USA

**Keywords:** carcinoid heart disease, hedinger syndrome, right-sided heart failure, tricuspid valve regurgitation, gastrointestinal carcinoid tumor

## Abstract

Carcinoid syndrome can cause desmoplastic reactions to nearby tissues. When it involves the heart, it causes carcinoid heart disease (CHD) or Hedinger syndrome and usually involves the right-sided heart valves, causing tricuspid insufficiency and pulmonary stenosis (TIPS) and eventually leading to right-sided heart failure. The management of patients with CHD is complex, as both the systemic malignant disease and the heart involvement have to be addressed. Its prompt diagnosis and early treatment is paramount as CHD is associated with increased morbidity and mortality. A 61-year-old Caucasian male with a recently diagnosed metastatic neuroendocrine tumor presented to the heart failure clinic with decompensated heart failure, anasarca, flushing and diarrhea. This case highlights the common clinical features of carcinoid syndrome, its cardiac manifestations and the pathophysiology underlying the manifestations and treatment decisions that involve addressing both systemic and cardiac manifestations.

## Introduction

Carcinoid heart disease (CHD) is a manifestation of valvular heart disease occurring in patients with neuroendocrine tumors. The pathophysiology of CHD involves serotonin metabolism as well as a variety of substances secreted by the neuroendocrine tumor itself. Previously, these tumors were known as carcinoid tumors; they are rare malignancies occurring with an incidence of 5.1/100,000 per year, most frequently arising out of the gastrointestinal tract (67.5%), most from small intestine, followed by the pulmonary system (25.3%) [1,2]. CHD or Hedinger syndrome is a dire complication in patients with carcinoid syndrome. About 25% of patients end up with cardiac involvement, which comprises right-sided heart valve degenerations that can be mixed-stenotic and regurgitative in pathology and consequently lead to heart failure symptoms, resulting in significant morbidity and mortality [3].

## Case presentation

We report a case of a 61-year-old Caucasian male with no past medical history who was referred by his oncologist to the heart failure clinic after auscultating an abnormal murmur. The patient had been generally healthy and had not sought any medical attention until he developed bright red stool six months ago; he underwent a colonoscopy that was normal. Unfortunately, one month ago prior to the presentation to the clinic, he started developing peripheral edema, which progressively worsened leading to rapid development of anasarca over the course of two weeks. A review of systems was positive for diarrhea, progressive fatigue, flushing and diffuse pruritis. He reported gaining 12 kg over the course of two months. He was unable to perform daily chores, but was able to take a shower by himself. Exertional dyspnea was seen after walking only two blocks; he also had nausea without vomiting and generalized fatigue. He had tried taking oral diuretics for a few days, but had had no improvement. He also reported swelling around his penis and scrotum. His diarrhea was adequately managed with loperamide.

Physical examination was remarkable for tachycardia of 101 beats per minute, tachypnea of 20 breaths per minute, with otherwise normal blood pressure and oxygen saturation. The patient was in mild respiratory distress and had been sitting up in bed with the bed elevated at 60°. He had anicteric sclera and no stigmata of cirrhosis. Skin examination revealed a diffusely erythematous blanching rash on his forehead, face and around his chest. His jugular venous pulse was at his earlobe and he had a positive Lancisi's sign. Cardiac examination was remarkable for regular tachycardic rhythm with normal S1 and S2. The patient had a loud holosystolic grade 3 out of 6 murmur at the left lower sternal border that was worsened by inspiration and leg raise, radiating to the axilla. He had no S3 gallops, no friction rub or clicks; lungs were clear to auscultation bilaterally and no rales or wheezes were noted. His abdomen was soft yet protuberant, mildly tender around the right upper quadrant and lower quadrants around the previous biopsy site. He had no hepatomegaly or splenomegaly on examination, and endorsed normoactive bowel sounds. He did have a positive hepatojugular reflux on examination. His extremities were warm to touch with profound tense woody edema up to the torso. Skin examination revealed warm diffuse blanching erythema from head to toe, including palms and soles; the nape of his neck was slightly darker. Neurological examination was nonfocal with grossly intact cranial nerves. The patient had a normal and steady gait with clear and fluent speech.

Lab values revealed reactive leukocytosis, synthetic liver dysfunction with elevated liver enzymes and mildly elevated coagulation parameters, consistent with congestive hepatopathy (Table 1). The patient had an elevated BNP with negative troponins and normal basic metabolic profile. He was found to have an elevated 24-hour urinary 5-hydroxyindoleacetic acid (5-HIAA) level and an elevated chromogranin A level. Niacin levels were within normal limits.

**Table 1:** Laboratory workup eGFR CKD-EPI Cr, estimated glomerular filtration rate using the Chronic Kidney Disease - Epidemiology Collaboration equation; HCO_3_, bicarbonate; SGOT (AST), serum glutamic-oxaloacetic transaminase (aspartate transaminase); SGPT (ALT), serum glutamic-pyruvic transaminase (alanine aminotransferase); INR, international normalized ratio; PT, prothrombin time; PTT, partial thromboplastin; MCV, mean corpuscular volume; MCH, mean corpuscular hemoglobin; MCHC, mean corpuscular hemoglobin concentration; RDW, red cell distribution width; Abs, absolute; BNP, B-natriuretic peptide; 5-HIAA, 5-hydroxyindoleacetic acid; H, high; L, low

	Patient’s values	Normal reference values
Complete metabolic profile		
Glucose	108 (H)	70-99 mg/dL
Blood urea nitrogen	9	6-23 mg/dL
Creatinine	0.7	0.6-1.4 mg/dL
eGFR CKD-EPI Creatinine 2021	105	≥90 mL/min/1.73 m^2^
Sodium	134	133-145 mEq/L
Potassium	4.5	3.3-5.1 mEq/L
Chloride	101	95-108 mEq/L
HCO_3_	22	21-30 mEq/L
Calcium	8.4	8.3-10.5 mg/dL
SGOT (AST)	18	0-40 IU/L
SGPT (ALT)	20	0-41 IU/L
Alkaline phosphatase	271 (H)	35-129 IU/L
Bilirubin, total	1.3 (H)	0-1.2 mg/dL
Total protein	6.1 (L)	6.4-8.3 g/dL
Albumin	3.0 (L)	3.5-5.2 g/dL
Globulin	3.1	2.1-3.7 g/dL
Albumin/globulin ratio	1.0	1.0-2.4
Complete blood count with differential		
White blood cell count	10.43	3.80-10.80 × 10^3^/uL
Red blood cell count	4.44	4.00-6.20 × 10^6^/uL
Hemoglobin	13.6 (L)	13.7-17.5 g/dL
Hematocrit	41.7	40.1-51.0%
MCV	93.9	79.4-98.4 fL
MCH	30.6	26.0-34.0 pg
MCHC	32.6	32.0-36.0 g/dL
RDW	15.5 (H)	11.6-14.4%
Platelet count	358	151-424 × 10^3^/uL
Immature granulocyte	0.3	0.0-1.0%
Neutrophil	75.9 (H)	34.0-72.0%
Lymphocyte	11.6 (L)	12.0-44.0%
Monocyte	9.6	0.0-12.0%
Eosinophil	2.0	0.0-7.0%
Basophil	0.6	0.0-2.0%
Abs immature granulocytes	0.03	0.0-0.10 × 10^3^/uL
Abs neutrophils	7.92 (H)	1.56-6.20 × 10^3^/uL
Abs lymphocytes	1.21	1.18-3.74 × 10^3^/uL
Coagulation studies		
Protime	15.0 (H)	11.8-14.2 s
INR	1.3 (H)	0.9-1.0
PTT	39.7 (H)	23.5-37.8 s
Cardiac biomarkers		
B-natriuretic peptide	101 (H)	<100 pg/mL
Troponin T fifth-generation	10	<19 ng/L
Neuroendocrine markers		
Urine 5-HIAA	486 (H)	<6 mg/24 hr
Plasma chromogranin A	3935 (H)	0-103 ng/mL

**Figure 1:**
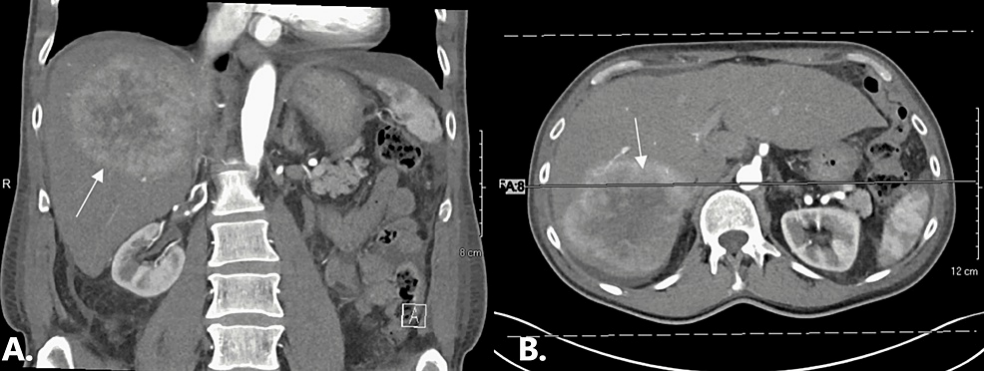
Abdomen computed tomography with liver protocol (A) Coronal view showing a 12.3 × 9.9 × 9.8 cm arterial enhancing mass with washout and pseudo-capsule centered in the posterior right hepatic lobe (arrow); (B) sagittal view showing the same enhancing mass in the posterior right hepatic lobe (arrow)

**Figure 2:**
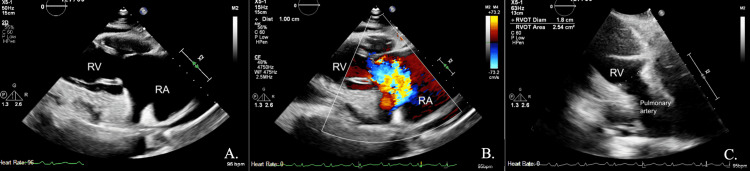
Transthoracic echocardiogram of the tricuspid valve RA, right atrium; RV, right ventricle (A) Right ventricular inflow view demonstrating retracted anterior and posterior leaflets of the tricuspid valve, resulting in non-coaptation at the end-systole. (B) Color flow Doppler image showing “wide-open” tricuspid regurgitation as a result of non-coaptation. (C) Right ventricular outflow tract showing a decreased diameter (1.8 cm) across the pulmonic valve, resulting in pulmonic stenosis (normal: 2.1-3.1 cm)

Prior to this presentation, he had been generally healthy and had been followed up annually by his primary care physician (PCP); he had never been hospitalized in the past. Given his ongoing diarrhea, his PCP ordered a computed tomography (CT) scan of the abdomen and pelvis that revealed masses to the liver and an enlarged mesenteric lymph node. Follow-up CT with the liver protocol revealed a 12.3 × 9.9 × 9.8 cm arterial enhancing mass in the right hepatic lobe, as well as five smaller masses in the right lobe, one mass in the left lobe, and a 3.3-cm mass in the mesentery in the right mid abdomen (Figure 1). Four days later, he underwent liver biopsy and pathology revealed a well-differentiated neuroendocrine tumor. He was referred to an oncologist who ordered an echocardiogram after detecting a murmur on auscultation. Echocardiogram revealed mildly decreased left ventricular function of 45% with flattening of the septal wall, severely dilated right ventricle with normal systolic function, non-coaptating tricuspid valve leaflets with severe (wide-open) regurgitation, as well as mild pulmonic stenosis (Figure 2).

**Figure 3:**
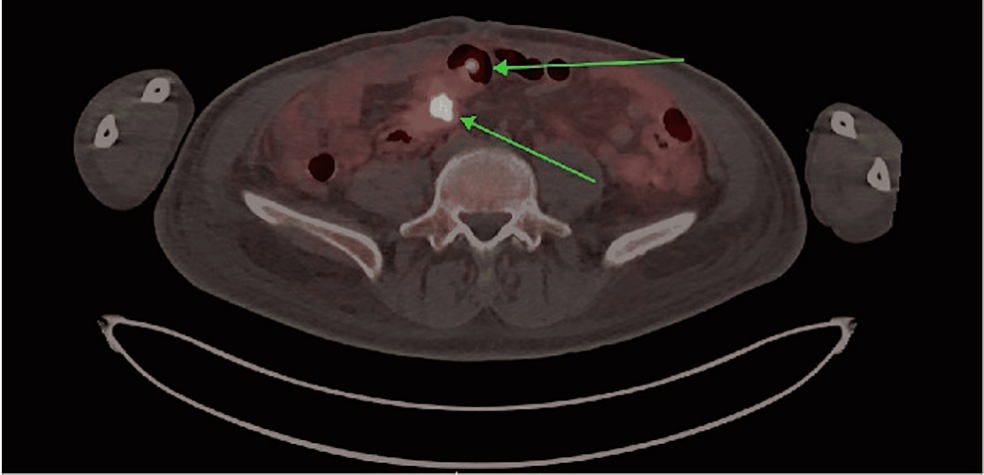
A PET/CT scan revealing a 3.3-cm focus (green arrows) of the upregulated somatostatin receptor in the small bowel consistent with a primary neuroendocrine tumor PET/CT, positron emission tomography/computed tomography

**Figure 4:**
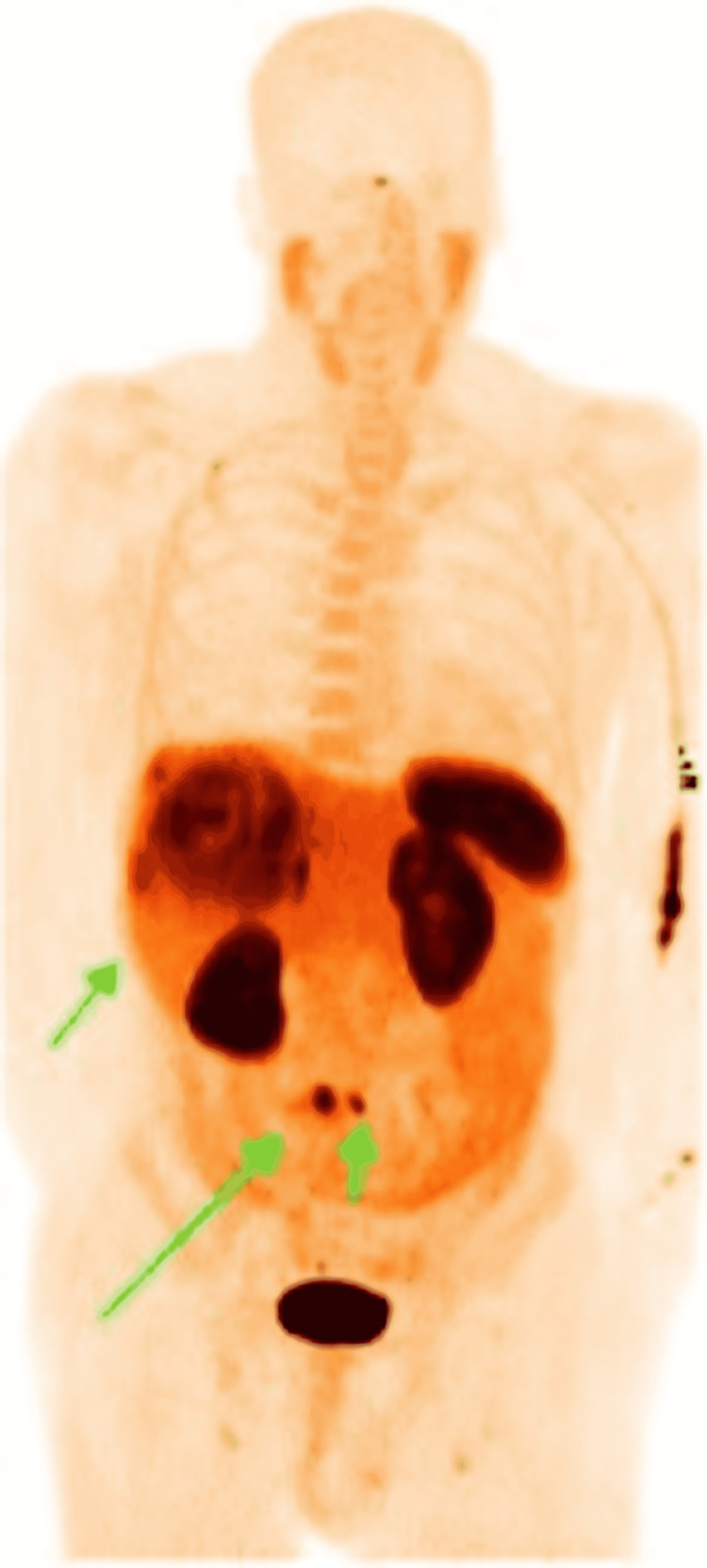
A PET/CT scan revealing several somatostatin-receptor-positive hepatic tumors in both lobes of the liver as well as a 3.3-cm mesenteric nodule (green arrows) PET/CT, positron emission tomography/computed tomography

During the hospitalization, he underwent a positron emission tomography (PET) scan revealing a focus of the upregulated somatostatin receptor in the small bowel, suspected to be the primary neuroendocrine tumor (Figure 3). Several somatostatin-receptor-positive hepatic tumors were noted, in both lobes of the liver, as well as a 3.3-cm mesenteric nodule, representing mesenteric metastasis (Figure 4). There was a focus of increased somatostatin receptors in the lateral right second rib, thought to represent an early bone marrow metastasis. After volume status optimization, the patient was started on somatostatin analogue treatment with close follow-up. He was discharged a few days later with an outpatient diuretic regimen with plans to move out of state for continuation of his care. Unfortunately, three months later, the patient presented to the emergency department with cardiogenic shock, prompting emergent valve replacement surgery, after which the patient developed postoperative mixed cardiogenic and distributive shock, not a candidate for extracorporeal membrane oxygenation (ECMO) given inappropriate vasculature. His course was complicated by mesenteric ischemia leading to severe lactic acidosis; given the dire prognosis, the family decided to pursue comfort measures.

## Discussion

Hedinger syndrome is a unique and unfortunate complication of carcinoid syndrome. More than half of CHD patients have had either a neuroendocrine tumor or carcinoid syndrome. This desmoplastic tumor generally involves the right side of the heart [4]. In fact, left-sided lesions are rare unless some form of atrial septal defect is present. Most commonly (in around 70% cases), such as in our patient, the tumor originates from the small bowel, followed by lung, colorectal, pancreas, and appendix; even ovarian origins have been described in some cases. However, in approximately 20% of the case presentations, the primary tumor location remains unknown [5]. Males are slightly more affected in a 3:2 male-to-female ratio with the mean age of diagnosis in the fifth or sixth decade of life.

Early in the course, clinical manifestation can be difficult to diagnose, as patients can be asymptomatic from various degrees of tricuspid and pulmonary valve disease. This is partly due to the low-pressure pulmonary circulation [6,7]. Early presentation involves symptoms of heart failure presenting as easy fatiguability and exertional shortness of breath. Then, as the desmoplastic tumor progresses, and the levels of serotonin increase, patients have regressively worsening right-sided heart failure with worsening dyspnea and anasarca. It is important to note that sometimes no symptoms or signs suggesting CHD are found despite the cardiac manifestation. A high degree of clinical suspicion is required to establish a timely diagnosis in these patients. There is an emerging body of evidence that points to the repeated stimulation of 5-hydroxytryptamine (5-HT)-2B receptors that induces uncontrolled valve cell division [8,9]. Interestingly, dopamine agonist uses in patients with Parkinson’s disease (pergolide or cabergoline) have also been associated with the occurrence of similar valvular changes, pointing to this mechanism as an important step in pathogenesis [8,10].

Another important yet rare association with carcinoid syndrome is pellagra, which occurs as a result of niacin deficiency. Endogenous niacin production is depressed, as tumor cells divert the available tryptophan substrate metabolism towards serotonin production. The decreased availability of endogenous and exogenous niacin eventually results in the depressed tissue niacin levels responsible for the development of pellagra, leading to dermatitis, dementia, diarrhea, and even death. Oral or parenteral administration of niacinamide (vitamin B3) may be required for rapid clearing of the pellagrous dermatitis.

Treatment is based on the availability of serotonin analogues and on neuroendocrine cell expression of cell membrane serotonin (5-HT) receptors. Tumor debulking methods such as hepatic artery embolization and palliative cytoreductive surgery may improve the symptoms in carcinoid syndrome; they can make symptoms of heart failure better [11,12]. Unfortunately, there is not enough evidence to suggest that these interventions are beneficial in terms of CHD progression [13]. Management is focused on relieving symptoms of right-sided heart failure, including cautious diuresis and salt restriction. However, in advanced right ventricular failure, these measures become ineffective and surgical valve replacement or balloon valvuloplasty may be the only option for heart failure symptom relief [14].

## Conclusions

Hedinger syndrome or carcinoid heart disease is a rare complication of advanced neuroendocrine tumors and is associated with increased morbidity and mortality. The pathophysiology in the development of CHD remains complex, but serotonin plays a pivotal role in the pathological process of valve destruction and dysfunction. Early recognition and surgical interventions, even before advanced heart failure consultation has occurred, may improve the outcomes in these patients. Our understanding of the molecular mechanism underlying the disease has increased significantly over the last decade, and pharmacotherapy designed for targeting the fibrosis in CHD may lead to the development of appropriate novel targets for the desmoplastic effect of the tumor. Patients should be managed in specialized centers by a multidisciplinary team including oncologists, advanced heart failure specialists, and cardiothoracic surgeons with experience in the treatment of this condition. Results of ongoing clinical trials may reveal further improvements in diagnosis and treatment.
